# Identification of a novel mutation of FGFR3 gene in a large Chinese pedigree with hypochondroplasia by next-generation sequencing: A case report and brief literature review

**DOI:** 10.1097/MD.0000000000014157

**Published:** 2019-01-25

**Authors:** Guixiang Yao, Guangxin Wang, Dawei Wang, Guohai Su

**Affiliations:** a Institute of Translational Medicine, Jinan Central Hospital Affiliated to Shandong University; b Cheeloo College of Medicine, Shandong University, Jinan, Shandong; c Department of Biomedical Sciences, City University of Hong Kong, Hong Kong SAR, China.

**Keywords:** c.1052C>T, FGFR3 gene, hypochondroplasia, next-generation sequencing

## Abstract

**Rationale:**

Hypochondroplasia (HCH) is the mildest form of chondrodysplasia characterized by disproportionate short stature, short extremities, and variable lumbar lordosis. It is caused by mutations in fibroblast growth factor receptor 3 (*FGFR3*) gene. Up to date, at least thirty mutations of *FGFR3* gene have been found to be related to HCH. However, mutational screening of the *FGFR3* gene is still far from completeness. Identification of more mutations is particularly important in diagnosis of HCH and will gain more insights into the molecular basis for the pathogenesis of HCH.

**Patient concerns:**

A large Chinese family consisting of 53 affected individuals with HCH phenotypes was examined.

**Diagnoses:**

A novel missense mutation, c.1052C>T, in *FGFR3* gene was identified in a large Chinese family with HCH. On the basis of this finding and clinical manifestations, the final diagnosis of HCH was made.

**Interventions:**

Next-generation sequencing (NGS) of DNA samples was performed to detect the mutation in the chondrodysplasia-related genes on the proband and her parents, which was confirmed by Sanger sequencing in the proband and most of other living affected family members.

**Outcomes:**

A novel missense mutation, c.1052C>T, in the extracellular, ligand-binding domain of FGFR3 was identified in a large Chinese family with HCH. This heterozygous mutation results in substitution of serine for phenylalanine at amino acid 351 (p.S351F) and co-segregates with the phenotype in this family. Molecular docking analysis reveals that this unique *FGFR3* mutation results in an enhancement of ligand-binding affinity between FGFR3 and its main ligand, fibroblast growth factor 9.

**Lessons:**

This novel mutation is the first mutation displaying an increase in ligand-binding affinity, therefore it may serve as a model to investigate ligand-dependent activity of FGF-FGFR complex. Our data also expanded the mutation spectrum of *FGFR3* gene and facilitated clinic diagnosis and genetic counseling for this family with HCH.

## 1 Introduction

Hypochondroplasia (HCH, MIM #146000) is the mildest form of fibroblast growth factor receptor 3 (FGFR3) chondrodysplasia group with an incidence of about 1 in 50,000.^[1,2]^ It is inherited in an autosomal dominant manner. HCH has a broader spectrum of phenotypes that occasionally overlap with those patients with achondroplasia (ACH, MIM #100800), another type of skeletal dysplasia with short-limbed dwarfism, and normal individuals of short stature.^[3,4]^ Its clinical and radiographical features include disproportionate short stature, rhizomelic shortening of the extremities, short fingers, macrocephaly, flared metaphyses, variable lumbar lordosis, progressive narrowing of interpediculate distance in the lumbar vertebrate, short and squared ilia, and shortened tubular bones and short femoral necks.^[5,6]^ Lack of trident hand, normal facies, and increased head circumference usually help distinguishing HCH from ACH.^[7–9]^

HCH is caused by mutations in *FGFR3* gene which maps to chromosome 4p16.3. This gene belongs to the tyrosine kinase receptor family that includes 4 known members (FGFR 1–4). FGFR3 is composed of 1 extracellular, ligand-binding domain including 3 immunoglobulin-like loops (Ig I–III), 1 hydrophobic transmembrane (TM) domain, and 2 cytoplasmic TK sub-domains TK1 and TK2 that are responsible for the catalytic activity.^[10]^ It is expressed during skeletal growth and endochondral ossification and plays an important role in the regulation, proliferation, differentiation, as well as other processes involved in growth and development.^[11]^ Up to date, at least 30 mutations have been reported to be related to HCH according to the Human Gene Mutation Database (HGMD, http://www.hgmd.org/), Pubmed, Embase, and Web of science. However, mutational screening of the *FGFR3* gene is still far from completeness. Identifying more novel mutations will gain more insights into the molecular basis for the pathogenesis of HCH.

With the development of new sequence technology, next-generation sequencing (NGS) has recently been used as an alternative approach to more traditional methods in the clinical practice including genetic diagnosis, family genetics counseling, and prenatal diagnostic testing.^[12]^ NGS has many advantages which can not only to produce massive amounts of data in parallel but also to measure each base pair to an unprecedented depth, which greatly reduces the time and cost of sequencing each sample at each locus.^[13]^

In this study, we described the clinical and radiographical manifestations of a large Chinese Han family with 53 affected individuals by HCH. Then, we used an approach based on targeted gene capture and NGS in this family and identified a novel missense mutation, c.1052C>T (S351F), in the extracellular, ligand-binding domain of FGFR3, which is outside the common mutation hot spot of this condition. Furthermore, in silico analysis was performed on this gain of function mutation. Identification of this novel mutation provides new insights into the molecular basis for the pathogenesis of HCH and assists early diagnosis.

## 2 Patients and methods

### 2.1 Proband and family investigation

**Figure 1:**
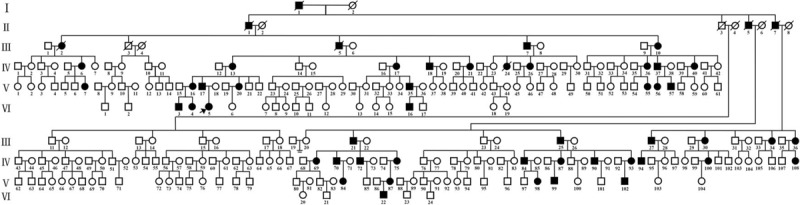
Pedigree of a large Chinese Han family with HCH (The proband is indicated with an arrow). HCH = hypochondroplasia.

The pedigree is shown in Figure 1. The proband (VI-5) was a 7-year and 8-month-old Chinese Han girl, who was born at full term after an uncomplicated pregnancy and delivery. At birth, she weighed 3.1 kg and her length was 50.5 cm. She was referred to hospital because of her short stature. The proband has not received any treatment up to the present.

The family history investigation shows that there were 52 other affected individuals in this Chinese Han family. All available individuals with a positive history underwent a full physical examination, including height, intelligence, and head circumference. Some family members had skeletal X-rays.

This study was approved by the research ethics committee of Jinan Central Hospital Affiliated to Shandong University. Informed consents were obtained from all subjects or their legal guardians. The parents of proband have provided informed consent for publication of the case.

### 2.2 Methods

#### 2.2.1 Targeted sequence capture and NGS

NGS was performed on the proband and her parents (VI-5; V-17; V-18).

2 mL of peripheral blood were collected and then preserved in anticoagulation tubes. Genomic DNA was isolated from peripheral whole blood using TIANamp Blood DNA Kit (Tiangen Biotech Beijing Co. LTD., China). After the DNA extraction, target sequences were enriched by using customized capture probes chips (Illumina, San Diego, CA), which included 10 genes (*FGFR3*, *COMP*, *BMPR1B*, *ARSE*, *AGPS*, *ACP5*, *GDF5*, *COL2A1*, *TRIP11,* and *SLC26A2* gene) that are associated with chondrodysplasia. DNA probes were designed for exons and flanking intron sequences (-20 base pairs). 1 μg genomic DNA was fragmented into 200 to 300 bp length by Covaris Acousitic System. The DNA fragments were then processed by end-repairing, A-tailing and adaptor ligation, a 4-cycle pre-capture PCR amplification, targeted sequences capture. Captured DNA fragments were eluted and amplified by 15 cycle post-capture PCR. The final products were sequenced with 150-bp paired-end reads on Illumina HiSeq X Ten platform according to the standard manual.

The clean short-reads were mapped to human genome (hg19) using BWA software (http://sourceforge.net/projects/bio-bwa/). SOAP snp software (http://soap.genomics.org.cn/) and SAM tools Pileup software (http://sourceforge.net/projects/samtools/) were used to detect single nucleotide variants (SNPs) and small insertions and deletions. Variants were annotated by ANNOVAR software, which is freely available at http://www.openbioinformatics.org/ annovar/. Variants were interpreted according to the American College of Medical Genetics and Genomics (ACMG) recommended standard.^[14,15]^

#### 2.2.2 Sanger sequencing

To validate true positive novel mutations identified by NGS, Sanger sequencing was performed to determine the presence or absence of this variant in the proband, most of other living affected family members, a part of unaffected family members (III-11; III-14; III-15; III-23; III-31; IV-2; IV-10; IV-14; V-3; V-9; V-10; V-37; V-38; V-42; V-64; V-72; V-73; V-101; VI-17; VI-20; VI-21; VI-24) and 50 unrelated healthy controls.

The specific PCR primers (forward primer 5^’^-ACCTGGGACAGAGGACTCGC-3^’^, reverse primer 5^’^- TGGAGGGTCTCGCAGTCAGT -3^’^) were used for the amplification of target gene site based on the reference sequences of human genome from GenBank in NCBI (NM_000142.4). PCR cycling was performed on a DNA thermal cycler (Gene Amp 9700, Perkin-Elmer, USA) with 2 × Hotstart Taq PCR Mastermix kit (Tiangen Biotech Beijing Co. LTD., China). In a 50 μL reaction mix, 300 ng of genomic DNA were used with 2.0 μL of each primer (10 μmol/L), and 25 μL of 2 × PCR Mastermix. Genomic DNA was first denatured at 94°C for 3 min, followed by 31 cycles of 94°C for 35 s, 60°C for 35 s, and 72°C for 50 s. The PCR products were extended at 72°C for 5 min. The products were gel-purified with an agrose gel DNA purification kit (Tiangen Biotech Beijing Co. LTD., China), and the purified PCR products were sequenced using the forward and reverse primers. Automated sequencing was performed at both ends on an ABI 377 automatic sequencer.

#### 2.2.3 In silico analysis

Deleterious effects of the detected novel mutation on protein function were predicted using 2 web-based tools: Sorting Intolerant from Tolerant (SIFT) and Polymorphism Phenotyping v2 (PolyPhen-2). SIFT (http://sift.bii.a-star.edu.sg/) is a sequences homology-based tool which presumes that the important amino acids are conserved in the protein family. Its results were expressed as SIFT scores which were classified as damaging (0.00–0.05), potentially damaging (0.051–0.10), borderline (0.101–0.20), or tolerant (0.201–1.00). PolyPhen-2 (http://genetics.bwh.harvard.edu/pph2/) is a web-based tool which predicts possible impact of an amino acid substitution on the structure and function of a human protein using straightforward physical and comparative considerations.^[16]^ Three possible outcomes of mutations were predicted by PolyPhen-2: probably damaging, possibly damaging or benign according to the score ranging from 0 to 1.

Three-dimensional structure modeling of wild and mutant FGFR3 was built by using the FR-t5-M^[17]^ and CIS-RR.^[18]^ The molecular docking analysis was performed with ZDOCK 3.0.2 (http://zdock.umassmed.edu/) to examine ligand-binding affinity between FGFR3 (wild and mutant) and its main ligand, fibroblast growth factor 9 (FGF9).^[19]^ The top 10 complex models were selected as candidates. The binding affinity was evaluated by ZDOCK score. PyMol (http://www.pymol.org/) was used to draw the structure models.

## 3 Results

### 3.1 Clinical data

**Figure 2:**
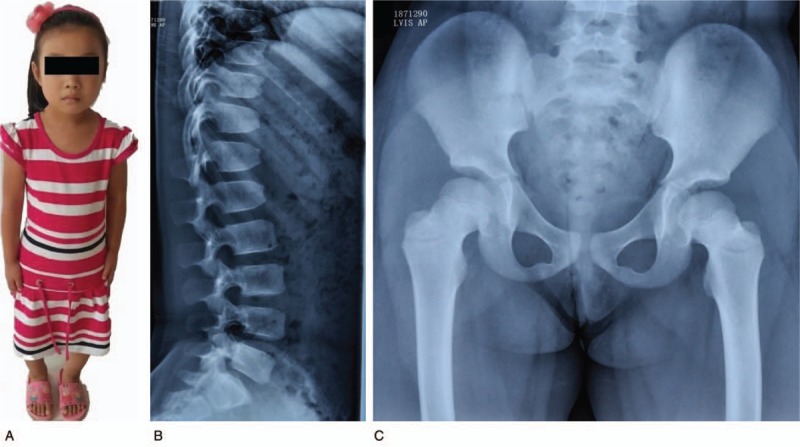
Clinical and radiologic findings of the proband at the age of 7 yr and 8 mo. A. Front view illustrated a disproportionate dwarf with normal face. B. Radiographs of front spine showed that interpedicular distance was slightly smaller at L-5 than at L-1. C. Radiographs of ilias revealed short iliac bones and short femoral necks.

The proband was a disproportionate dwarf with short-limbs, brachydactyly and slight genu varum (Fig. 2A) but she did not have trident hands. Her face is normal and she is of normal intelligence. Current weight was 23.1 kg (–0.3 SD), height 116.0 cm (–2.1 SD), her upper to lower segment ratio 1.32 and head circumference 52.2 cm. Her serum concentrations of alkaline phosphatase, insulin-like growth factor 1 (IGF-1), IGF-binding proteins, and thyroid hormone were normal.

Radiological studies revealed short limbs with slight brachydactyly, caudad narrowing of the interpediculate distance of the lumbar spine (Fig. 2B), short iliac bones and short femoral necks (Fig. 2C), short stubby tibias, mildly increased fibular lengths and genu varum. Her clinical and radiologic findings were consistent with clinico-radiologic criteria for HCH.^[8]^

The clinical features of living affected members with HCH in this large Chinese Han family are presented in the Table 1.

**Table 1:**
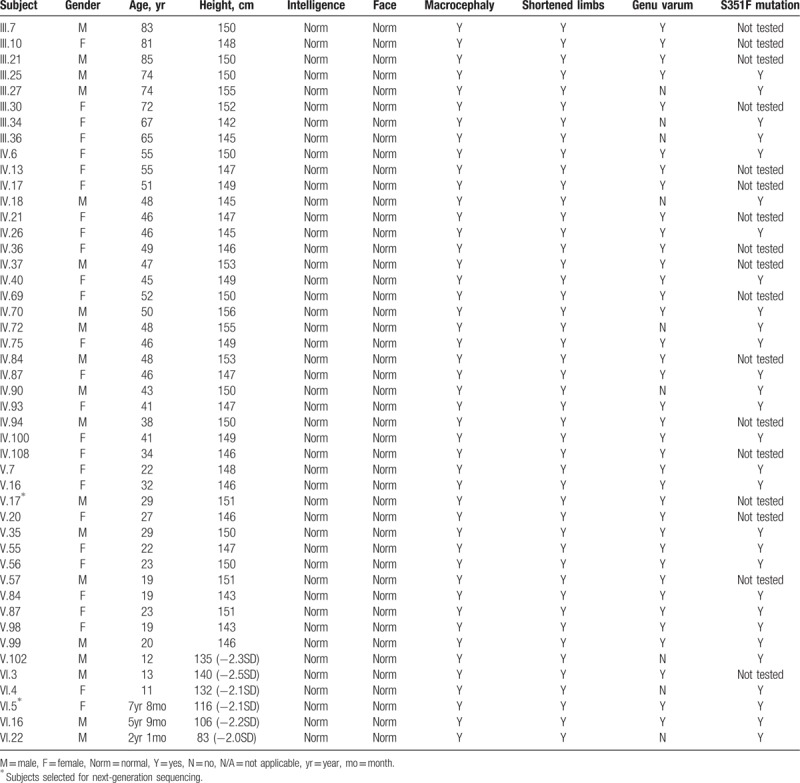
Clinical features of living members with HCH in a large Chinese Han family.

### 3.2 Mutation detection

**Figure 3:**
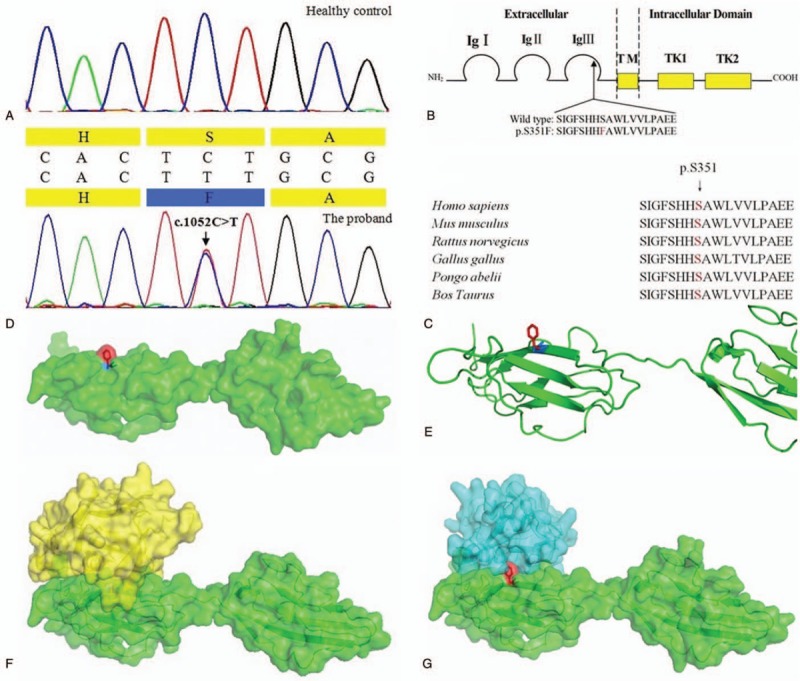
Sequencing results and in silico analysis of the gene mutation in our study. A. Partial Sanger sequencing diagram of *FGFR3* gene. A heterozygous mutation C1052T is shown by an arrow. B. The structure domains of FGFR3. The location of the novel mutation at protein level is shown by an arrow. C. Homology analysis of the *FGFR3* S351 mutation site (in red) in different species. D. Structure modeling of the wild and mutant FGFR3. Blue, red and green represent the wild-type region (S351), the mutant region (F351) and truncated structure of FGFR3, respectively. E. Detail View of FGFR3 structure modeling of the wild and mutant FGFR3. Blue, red and green represent the wild-type region (S351), the mutant region (F351) and truncated structure of FGFR3, respectively. F. The view of the wild FGFR3/FGF9 complex surface. The yellow color indicates the FGF9, and the green color indicates truncated structure of FGFR3. G. The view of the mutant FGFR3/FGF9 complex surface. The yellow color indicates the FGF9, the red color indicates mutant region and the green color indicates truncated structure of FGFR3. FGFR3 = fibroblast growth factor receptor 3.

In this study, 8 variants in *FGFR3* gene were detected by NGS. We then excluded those variants with an allele frequency more than 5% in the dbSNP data-base, 1000 human genome dataset, exome aggregation consortium (ExAC) and genome aggregation database (gnomAD). According to the detailed filtering criteria and analysis pipeline published before,^[20]^ a C to T transition at nucleotide position 1052 in the cDNA of *FGFR3* gene was identified in both the proband and her father. C1052T mutation was then confirmed by Sanger sequencing (Fig. 3A). This heterozygous mutation resides in the extracellular Ig domain III of FGFR3 (Fig. 3B) and results in the substitution of serine by phenylalanine (S3351F). It co-segregates with other affected individuals of the Chinese Han pedigree with HCH. No mutation at this site was found in 23 unaffected family members detected or in 50 unrelated healthy controls. According to the HGMD (http://www.hgmd.cf.ac.uk/docs/login. html), this heterozygous mutation is novel.

Our homology analysis of the S351F site in different animal species indicated that the position of this mutation was highly conserved (Fig. 3C), which supported the possibility that this mutation was pathogenic.

### 3.3 In silico analysis

The results of SIFT and PolyPhen-2 analysis of this mutation provided further evidence that the mutation is the cause of the clinical phenotype. This mutation is predicted to be deleterious with a SIFT score of 0.000 and possibly damaging with a Poly Phen-2 score of 0.935.

Three-dimensional structure of FGFR3 (S351F) was created by replacing S351 with F351. F351 has an influence on the surface of FGFR3 relative to S351, which causes this residue site to change from the sunk to the raised (Fig. 3D, E). The molecular docking was successfully completed between FGFR3 (the wild and the mutant) and its ligand FGF-9 respectively (Fig. 3F, G). Because the mutation S351F changes the FGFR3 interface, the mutant FGFR3 displayed stronger binding to FGF-9 compared to the wild FGFR3, which is further supported by TOP10 ZDock Score (F351 vs S351, 1244.6 ± 64.2 vs 1115.8 ± 37.3, *P* <.01).

## 4 Discussion

HCH is characterized by clinical manifestations with the average height of adults—146 cm in males and 138 cm in females. The diagnosis of HCH is suspected on clinical grounds of short stature, a height prediction inappropriate for the family, and in some cases by diminution of the pubertal growth spurt and confirmed by several distinctive radiologic findings, such as brachydactyly, metaphyseal flaring, shortening of the pedicles of the vertebrae, lack of increase in interpedicular distance between lumbar vertebrae L1 and L5, square iliae, short femoral necks, and short tubular bones.^[5,21–23]^

However, the diagnosis of HCH is hampered sometimes because severe forms of HCH may overlap clinically with ACH, especially in younger children, and even some HCH patients may be diagnosed with idiopathic short stature because of its less severe skeletal features than patients with ACH. In the present study, a very large HCH family with 53 affected members is reported. All patients have typical features of the HCH. There is small variable expressivity within this family, for example, most of them have genu varum, but small number of them doesn’t have this symptom. All affected members lack a trident hand and abnormal face that may help distinguish HCH from ACH. The radiologic findings of the proband and some affected family members show short limbs with slight brachydactyly, narrowing of interpedicular distance between lumbar vertebrae L1 and L5, short iliac bones and short femoral necks, which are consistent with radiologic criteria for HCH.

Human *FGFR3* gene was identified as the HCH gene in 1995.^[24,25]^ This gene consists of 19 exons and 18 introns,^[26]^ and encodes an 840-residue FGFR3, a trans-membrane receptor that belongs to FGFR superfamily (FGFRs) including FGFR1 to FGFR4.^[27,28]^ FGFRs are responsible for mediating the transmission of the intracellular signaling cascade generated when they bind to the fibroblast growth factors (FGFs), including 18 members (FGF1–FGF10 and FGF16–FGF23).^[7]^ The formation of the dimer FGF-FGFR induces receptor dimerization, leading to auto- and transphosphorylation, followed by controlled activation of specific signal transduction pathways and expression of selected target genes in developmental pathways, which seems to have a specific role as negative regulator of bone growth.^[29–31]^ FGFR-3 deficient mice had a marked increase in the length of the vertebral column and long bones as a result of enhanced and prolonged bone growth. Indeed, the known *FGFR3* gene mutations related to HCH are gain of function mutations with ligand-independent activation of the receptor that results in decreased inhibition of endochondral ossification.^[11]^

Up to date, at least thirty mutations have been reported to be related to HCH according to HGMD, Pubmed, Embase, and Web of Science. All mutations that were hitherto reported were listed in Table 2. About 60% of cases of HCH are due to mutations in the intracellular FGFR3-tyrosine kinase domain, such as N540K, I538 V, though mutations may present in every domain of FGFR3.^[21,40,41]^ In this research, by using NGS and Sanger sequencing, we identified a novel missense mutation (C1052T) resulting in S351F substitution in the extracellular domain Ig III in a very large Chinese family consisting of 53 affected individuals with classic HCH phenotypes.

**Table 2:**
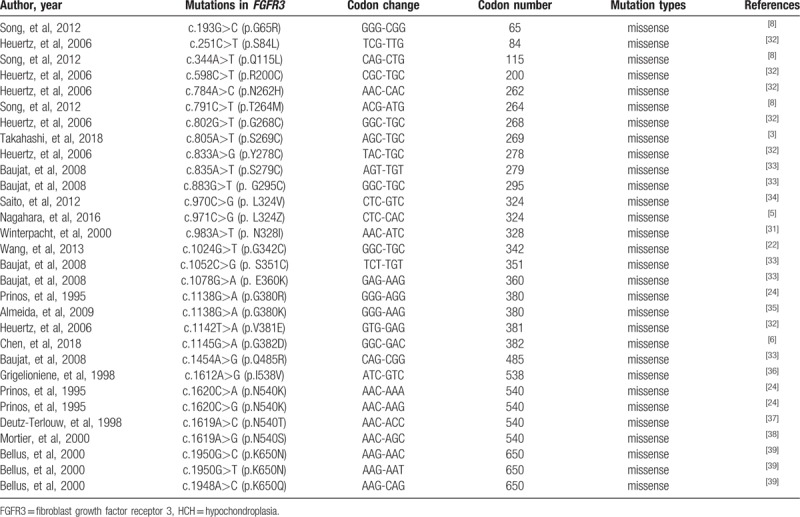
The summary of previously reported FGFR3 mutations causing HCH.

After searching the SNP database and the human gene mutation database, we found that c.1052C→T[p.S351F] was absent from these databases. We demonstrate that this family carries a novel heterozygous mutation based on the following evidences. First, the mutation is not present in the unaffected family members or in 50 unrelated healthy controls. Second, the mutation resides in a highly conserved region when comparing different animal species. Third, prediction of functional consequence shows that this mutation is deleterious with a SIFT score of 0.000 and possibly damaging with a Poly Phen-2 score of 0.935.

Thirty mutations reported previously affect different aspects of FGFR3 function. The common denominator among them was thought to be constitutive, ligand-independent activation of the receptor, leading to impaired chondrocyte differentiation.^[30]^ In this study, the S351F mutation is in the extracellular, ligand-binding domain and outside the intracellular domain TK1 or TK2 of FGFR3, without altering significantly 3-dimensional structure of TK1 or TK2 or disrupting normal folding of the IgIII loop. Nevertheless, this gain of function mutation displays an increase in ligand-binding affinity, therefore it may serve as a model to investigate ligand-dependent activity of FGF-FGFR complex.

Thus far, the phenotype/genotype correlation in patients with HCH is poor, though clinical heterogeneity has been associated with either different mutations in the *FGFR3* gene or with cases unlinked to the *FGFR3* gene. Ramaswami et al reported that HCH children with heterozygous N540K mutation were more severely affected than those without N540K mutation, and the former had a higher and larger forehead and shorter hands than the latter.^[21]^ Some studies showed that individuals with the FGFR3 N540K mutation may have an increased incidence of mild to moderate intellectual disability or learning disabilities.^[42]^ However, Song et al (1999) suggested that the clinical findings were similar in 2 groups of HCH with or without FGFR3 mutations, only the radiological findings of mesomelia of upper and lower limbs and, L1/L4 ratio in anterior–posterior and lateral view were more typical in HCH with FGFR3 mutations than in HCH without FGFR3 mutations.^[8]^ De Rosa et al described a 16-month-old male with N540K homozygous mutation in the FGFR3 gene who showed a more severe phenotype than the usual heterozygous HCH, which was even shorter than the heterozygous ACH.^[43]^ In our research, the main symptom (short stature) was mild in this HCH family with height of adults—145∼159 cm in males, 142∼152 cm in females and children, −2.0∼−2.5 SD in boys, −2.1SD in a girl, whether that arises from enhanced ligand-binding affinity or not should be further investigated.

## 5 Conclusion

In summary, we present evidence that S351F mutation represents a novel FGFR3 mutation in a large Chinese family with HCH. This gain of function mutation does not alter significantly 3-dimensional structure of TK1 or TK2 or disrupting normal folding of the IgIII loop. Nevertheless, this unique mutation displays enhanced FGF9-binding affinity, therefore it may serve as a model to investigate ligand-dependent activity of FGF-FGFR complex. Our data extend the mutation spectrum of *FGFR3* gene and have important implications for investigating ligand-dependent activation of the receptor and genetic counseling of the family.

## Acknowledgments

The authors thank the patients and their family members for their cooperation in this study.

The authors are grateful to Dr Carl E Stafstrom and Dr. Li-rong Shao, professor and assistant professor respectively at Department of Neurology, School of Medicine, Johns Hopkins University, USA, for proofreading the manuscript.

## Author contributions

**Data curation:** Guixiang Yao, Guangxin Wang.

**Formal analysis:** Guixiang Yao, Guangxin Wang.

**Funding acquisition:** Guohai Su.

**Investigation:** Guixiang Yao, Guangxin Wang.

**Project administration:** Guixiang Yao, Dawei Wang, Guohai Su.

**Resources:** Guangxin Wang, Dawei Wang.

**Software:** Dawei Wang.

**Supervision:** Guohai Su.

**Writing – original draft:** Guixiang Yao, Guangxin Wang.

**Writing – review & editing:** Guohai Su.
